# Can sibutramine alter systemic blood pressure in obese patients? Systematic review and meta-analysis

**DOI:** 10.1590/S1516-31802008000600010

**Published:** 2008-11-06

**Authors:** Hernani Pinto de Lemos, Álvaro Nagib Atallah, André Luis Alves de Lemos

**Keywords:** Obesity, Body mass index, Overweight, Hypertension, Anti-obesity agents, Obesidade, Índice de massa corporal, Sobrepeso, Hipertensão, Agentes anti-obesidade

## Abstract

**CONTEXT:**

Systemic arterial hypertension is part of the metabolic syndrome resulting from obesity.

**OBJECTIVE:**

To evaluate the effect of sibutramine on overweight and obese patients’ blood pressure through a systematic review.

**METHODS:**

All the studies included needed to be randomized controlled trials. The methodological quality of the selected trials was assessed using the criteria described in the Cochrane Handbook. The participants were overweight and obese patients; the intervention was sibutramine compared with placebo. The primary outcome measurement was systolic and diastolic blood pressure and the secondary measurement was blood pressure. Studies were identified by searching the following sources: Literatura Latino-Americana e do Caribe em Ciências da Saúde (Lilacs), Medline, Cochrane reviews, manual searches, personal communication and contact with the pharmaceutical industry. There were no language, date or other restrictions. Data collection and extraction was performed by two reviewers, who independently obtained the full articles of all eligible papers.

**RESULTS:**

Three meta-analyses were produced: 1) systolic blood pressure outcome (eight studies) did not show statistical significance between sibutramine and placebo: weighted mean difference (WMD) 1.57, confidence interval (CI) -0.03 to 3.18; 2) diastolic blood pressure outcome (ten studies) did not show statistical significance between sibutramine and placebo: WMD 1.13, CI -0.49 to 2.76; 3) blood pressure outcome (two studies) also did not show statistical significance between the groups: relative risk (RR) 0.69, CI 0.07 to 7.01.

**CONCLUSIONS:**

The meta-analyses presented in this systematic review show that sibutramine does not have a statistically significant effect on blood pressure, compared with placebo.

## RESUMO

**CONTEXTO:**

Hipertensão arterial sistêmica é parte da síndrome metabólica relacionada à obesidade.

**OBJETIVO:**

Avaliar o efeito da sibutramina na pressão arterial sistêmica de pacientes com sobrepeso e obesos através de uma revisão sistemática.

**MÉTODOS:**

Tipos de estudos: todos os estudos devem ser controlados e aleatórios. A qualidade metodológica dos estudos selecionados foi acessada usando os critérios descritos no Cochrane Handbook; participantes: pacientes com sobrepeso e obesos; intervenção: sibutramina comparada com placebo. Desfechos primários: pressão arterial sistólica e diastólica; secundário: pressão arterial. Estratégia de busca: os estudos foram identificados das seguintes fontes: Literatura Latino-Americana e do Caribe em Ciências da Saúde (Lilacs), Medline (Medical Literature Analysis and Retrieval System Online), Cochrane reviews (fontes eletrônicas), manuais, comunicação pessoal e contato com indústria farmacêutica, sem limites. Coleta de dados: dois revisores independentemente obtiveram os artigos completos de todas as publicações elegíveis.

**RESULTADOS:**

Três metanálises foram realizadas: no desfecho pressão arterial sistólica, com oito estudos, não houve significância estatística para a diferença entre a sibutramina e o placebo, WMD (weighted mean difference) 1.57, intervalo de confiança (IC) de -0,03 a 3,18; no desfecho pressão arterial diastólica, com 10 estudos, também não houve significância estatística na diferença entre a sibutramina e o placebo, WMD 1.13, IC de -0,49 a 2,76) e no desfecho pressão sangüínea com somente dois estudos, também não foi demonstrada diferença estatisticamente significante entre os grupos experimental e controle, risco relativo de 0,69, IC de 0,07 a 7,01.

**CONCLUSÃO:**

O resultado das metanálises apresentadas nesta revisão mostra que a sibutramina utilizada em pacientes obesos, quando comparada ao placebo, não tem efeito estatisticamente significante na pressão arterial.

## INTRODUCTION

The prevalence of obesity and its comorbidities has been increasing all over the world.^1^ Abdominal or visceral obesity is closely related to hypertension, glucose intolerance, hypertriglyceridemia and hyperinsulinemia, thus resulting in the so-called "metabolic syndrome", with increased risk of cardiovascular disease.^2,3^ Hypotheses for the pathophysiology of arterial hypertension in obese patients have been proposed. One of them is that hyperinsulinemia secondary to insulin resistance leads to greater sympathetic activity and to renal sodium retention, which possibly accounts for the increase in pressure levels.^4,5^ Another hypothesis is that there is an association between arterial hypertension in obese patients and the mechanical compression of the renal parenchyma by visceral fat. This would lead to hyperactivation of the renin-angiotensin-aldosterone system, high sodium reabsorption and subsequent elevation of blood pressure by a mechanism independent from insulinemia.^6,7^

Sibutramine is a tertiary amine that was initially developed as an antidepressant medication. Subsequent studies showed that the drug had a significant effect on weight loss due to its satietogenic and calorigenic effects.^8,9^ Use of sibutramine is associated with increased satiety scores and lack of decline in 24-hour energy expenditure,^8,10^ which thereby induces weight loss. Sibutramine blocks serotonin, dopamine and noradrenaline uptake,^11^ and the presence of high adrenergic activity may interfere with the benefits resulting from weight loss and increase the systolic and diastolic blood pressure and pulse rate. The prevalence of obesity is increasing and, because sibutramine is increasingly prescribed, better understanding of its effects is required, particularly in relation to blood pressure. The best kind of analysis in this respect is certainly a systematic review of all eligible studies that have previously been produced.

## METHODS

This was a systematic review using the Cochrane methodology. Studies were identified from the following sources: Literatura Latino-Americana e do Caribe em Ciências da Saúde (Lilacs), Medline, Cochrane reviews, manual searches, personal communication and contact with the pharmaceutical industry. There were no language, date or other restrictions. All the studies needed to be randomized controlled trials.

The methodological quality of the selected trials was assessed using the criteria described in the Cochrane Collaboration Handbook.^12^ These criteria were based on evidence of strong relationships with regard to the potential for bias in the results and allocation concealment. For the purpose of the analysis in the present review, trials were included if they met criterion A or criterion B in the Handbook. Criterion A represented a low risk of bias, interpreted such that the plausible bias would be unlikely to seriously alter the results. Its relationship to individual criteria was that all of the criteria were met. Criterion B represented a moderate risk of bias, interpreted such that the plausible bias would raise some doubt about the results. Its relationship to individual criteria was that one or more of the criteria were only partly met.^13^

Dichotomous outcomes were analyzed by calculating relative risks (RR) for each trial. The uncertainty in each result was expressed using confidence intervals (CI). Continuous outcomes were analyzed according to the differences in mean treatment effects and their standard deviations. A random-effects model was used for the meta-analyses.^14,15^

## RESULTS

### Systolic blood pressure (SBP)

**Figure 1:**
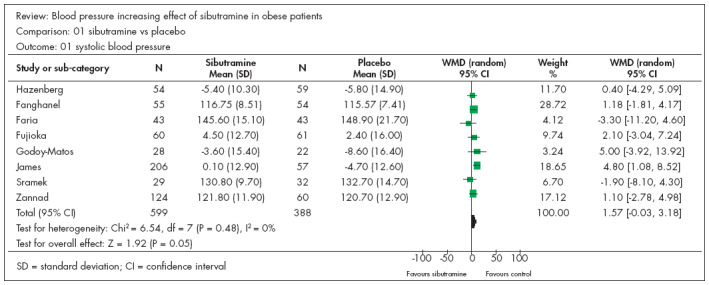
Systolic blood pressure and sibutramine in obese patients.

Eight studies^9,16-22^ presented continuous data that could be analyzed in relation to the outcome of systolic blood pressure (SBP). There were 599 participants in the sibutramine group and 388 in the control group. Only one study^9^ showed a statistically significant difference in relation to the control group. The resulting meta-analysis did not find any statistical differences between the groups, with a weighted mean difference (WMD) of 1.57 and CI from -0.03 to 3.18 (Figure 1). Only one study^19^ was included in relation to this outcome. It was composed of diabetic patients among whom separate analysis did not demonstrate any statistically significant difference between sibutramine and placebo. In relation to this outcome, we also found one study on patients with hypertension that was controlled with beta-blockers.^21^ We made a separate analysis on this study and found that it did not show statistical significance for any group. Removing this study from the meta-analysis did not modify the end result. The meta-analysis on these last two studies^16,18^ began with participants presenting mean SBP greater than 140 mmHg, and none of them showed increased statistical significance with regard to SBP.

### Diastolic blood pressure (DBP)

**Figure 2:**
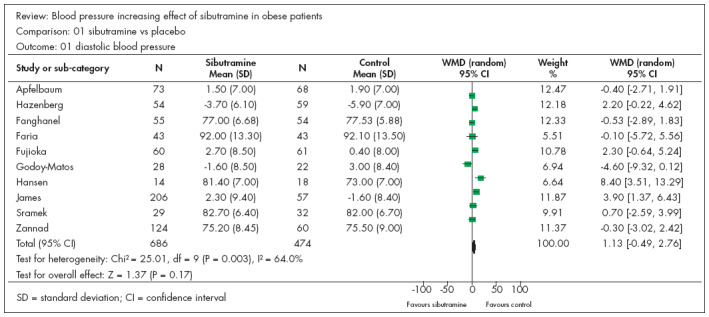
Diastolic blood pressure and sibutramine in obese patients.

**Figure 3:**
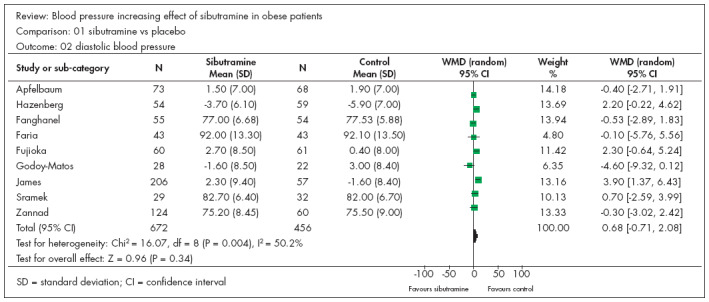
Diastolic blood pressure, as in Figure 2 but without the study by Hansen et al.

Ten studies^8,9,16-23^ presented continuous data that could be analyzed in relation to diastolic blood pressure (DBP). There were 686 participants in the sibutramine group and 474 in the control group. Two studies^8,9^ produced results favoring the control group, while the others did not show any statistical significance. The resulting meta-analysis did not show any statistical difference between the groups, with WMD of 1.17 and CI from -0.49 to 2.76 (Figure 2). Figure 2 indicates heterogeneity, with I^2^ = 64%. We made several analyses, removing studies one by one until we arrived at I^2^ = 50.2% (Figure 3), which was achieved by withdrawing the study by Hansen et al.^8^ (Figure 3). We investigated the reason for this and noticed that this study was the only one in this systematic review in which there was no dietary restriction. This observation confirms the concept of the importance of diet, independent of the drugs used. In the meta-analysis relating to this outcome, two studies^16,18^ began with participants whose mean DBP was greater than 90 mmHg, and neither of them increased the statistical significance of DBP.

The meta-analysis relating to this outcome also included one study on diabetics^19^ and another on patients with hypertension that was controlled with beta-blockers^21^ (the same study that was included in relation to the preceding outcome). We carried out the same procedure, removing each study from the meta-analysis, but did not find any statistically significant differences in the resultant.

One study^20^ that was included in relation to both the SBP and the DBP outcomes had been conducted among adolescents, aged 14-17 years. Because of the low prevalence of arterial hypertension in this age group, we removed this study from the meta-analysis. This did not modify the results, which continued not to present statistical significance for any group.

### Blood pressure (BP)

**Figure 4:**
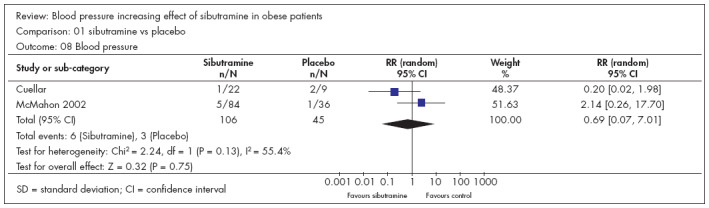
Blood pressure and sibutramine in obese patients.

The outcome of blood pressure (BP) was presented in two studies with dichotomous data.^24,25^ There were 106 participants in the sibutramine group and 45 in the control group. Neither of these studies showed statistical significance. Likewise, neither did the meta-analysis: RR 0.69 and CI from 0.07 to 7.01 (Figure 4).

Other studies could not be included in the meta-analysis because the statistical data extracted from them were either continuous variables or single dichotomous variables. One study^26^ with continuous data relating to the outcome of blood pressure made a comparison between sibutramine and placebo and did not find any statistically significant difference between the groups: WMD -0.10 and CI from -1.24 to 1.04. One study,^27^ with the outcome of systolic blood pressure presented dichotomous data, with 142 participants in the sibutramine group and 69 in the control group. The resultant analysis did not show any statistically significant difference between the groups: RR 1.30 and CI from 0.64 to 2.64. This same study^27^ presented the outcome of diastolic blood pressure and did not find any statistically significant significance between the sibutramine and control groups: RR 0.12 and CI from 0.01 to 1.07.

## DISCUSSION

The studies were analyzed with regard to the variables described below.

### Sibutramine doses

– 10 mg: six studies^16-18,20,22,23^ used this dose and the results did not show statistical significance for any group;
– 15 mg: one study^8^ used this fixed dose throughout the study. No statistical difference in systolic blood pressure was found (the author did not give data on the statistical analyses). However, there was a statistically significant difference favoring the control group, in relation to diastolic blood pressure (CI = 3.51 to 13.29);
– Increasing doses, from 5 mg to 20 mg: two studies^19,21^ used this scheme and neither of them showed any statistical significance favoring either the experimental or the control group;
– Increasing doses, depending on weight loss, up to 20 mg: only one study used this strategy,^9^ and it was the only one that found statistical significance for the control group, in relation to both systolic blood and diastolic blood pressure. This variable (sibutramine dose used), did not show any trends in any of the studies, except in one^9^ in which non-response (i.e. lack of weight loss) gave rise to a gradual increase in sibutramine dose, thereby causing increases in systolic and diastolic blood pressures.

### Body mass index (BMI)

This variable was capable of influencing the result, depending on the inclusion criteria limits imposed: BMI less than 30 kg/m²: six studies;^16,19,21,25-27^ BMI greater than 40 kg/m^2^:, three studies;^9,18,20^ BMI up to 50 kg/m^2^:, one study.^18^ Despite the different inclusion criteria relating to BMI, the mean for all participants was between 33 and 35 kg/m^2^, except for one study^18^ that had a higher mean of 39.5 kg/m^2^ and another^26^ that had a smaller mean of 30.8 kg/m^2^.

Although the inclusion criteria relating to BMI were different in the studies included in the meta-analyses for the SBP and DBP outcomes, the BMI values were, in reality, about average in most of the studies and therefore did not influence the results.

In one study^21^ the participants had arterial hypertension that was controlled with beta-blockers, whereas in another study,^19^ the use of beta-blockers was an exclusion criterion. In the first of these two studies, there was no statistical significance for any group, while in the second study there was statistical significance for the control group. This detail suggests the possibility that concomitant use of adrenergic blockers could minimize or annul the harmful effects of sibutramine use on the cardiovascular system.

In the worldwide literature, we did not find any systematic review with Cochrane methodology that encompassed a reasonable number of statistically representative studies for the main outcomes included in our systematic review. In 2004, a systematic review on the metabolic effects of sibutramine^28^ was published without any mention of arterial pressure (outcomes: weight reduction and maintenance of weight loss; effects relating to glycemic control and type 2 diabetes; effects on lipids, non-alcoholic fatty liver disease, serum uric acid levels, adipocytokines and C-reactive protein; plasma fibrinogen levels; polycystic ovary syndrome; and plasma homocysteine levels). The 2004 study was a review with important objectives, but without considering a common and routine outcome like blood pressure.

A systematic review evaluating the long-term efficacy of anti-obesity agents (orlistat and sibutramine) was published in the Cochrane Library in 2003,^29^ and included four sibutramine studies. The results showed that orlistat caused gastrointestinal side effects, while sibutramine was associated with small increases in blood pressure. Four years later, this review was improved with more studies, with the addition of another drug, and it was published in the British Medical Journal.^30^ It had the aim of summarizing the long-term efficacy of anti-obesity drugs (orlistat, rimonabant and sibutramine) in relation to weight reduction and changes in cardiovascular risk factors (blood pressure, lipid profile and HbA1c). Five sibutramine studies were included in the final review. Compared with placebo, sibutramine increased systolic blood pressure by 1.7 mmHg and diastolic blood pressure by 2.4 mm Hg. This systematic review reported that studies enrolling patients with diabetes reported slightly smaller weight losses with orlistat and rimonabant than with sibutramine. These two systematic reviews^29,30^ included studies that compared drugs with placebos but did not make comparisons between drugs. In fact, these reviews systematically assessed each drug and concluded by making a comparison between the drugs. This situation created a bias because, although these studies were of good methodological quality, the inclusion and exclusion criteria for each study were in accordance with the pharmacological properties of each drug and these properties were totally different for each drug.

Recently, in March 2008, a systematic review^31^ on obese patients with essential hypertension who received calorie-restricted diets or orlistat or sibutramine was published. All the groups were compared with placebo control groups. Four studies using sibutramine were included. One meta-analysis was produced from two studies for the outcome of diastolic blood pressure, and this showed statistical significance favoring the control group, with WMD of 3.16 and CI from 1.40 to 4.92.

The long-term nature of some studies makes it important to evaluate many outcomes such as maintenance of weight loss and biochemical changes due to dyslipidemia, diabetes etc. There is no need for long-term observation, for significant changes in blood pressure due to sibutramine to be seen. The study with the shortest follow-up in the present systematic review had a duration of three months, and this was enough time for the patients’ blood pressure to be changed through sibutramine use. Therefore, our results differ from the systematic reviews published previously, probably because of the larger number of studies with higher statistical power in the present review.

## CONCLUSIONS

The result from the studies presented in this systematic review showed that sibutramine did not have any statistically significant effect on blood pressure, in comparison with placebo.
